# SERPINA1 Gene Promoter Is Differentially Methylated in Peripheral Blood Mononuclear Cells of Pregnant Women

**DOI:** 10.3389/fcell.2020.550543

**Published:** 2020-09-03

**Authors:** John Charles Rotondo, Lucia Oton-Gonzalez, Rita Selvatici, Paola Rizzo, Rita Pavasini, Gianluca Calogero Campo, Carmen Lanzillotti, Chiara Mazziotta, Monica De Mattei, Mauro Tognon, Fernanda Martini

**Affiliations:** ^1^Department of Medical Sciences, University of Ferrara, Ferrara, Italy; ^2^Department of Morphology, Surgery and Experimental Medicine, University of Ferrara, Ferrara, Italy; ^3^Cardiology Unit, Azienda Ospedaliera Universitaria di Ferrara, Ferrara, Italy

**Keywords:** SERPINA1, Alpha1-AntiTrypsin, methylation, epigenetics, peripheral blood mononuclear cell, pregnancy

## Abstract

SERine Protein INhibitor-A1 (SERPINA1) is an inducible blood cell gene coding for alpha1-antitrypsin (AAT), a plasma protease inhibitor whose circulating levels are raised during inflammation, infection and advanced pregnancy. DNA methylation has been suggested to play a role in SERPINA1 gene expression regulation in peripheral blood mononuclear cells (PBMCs). The methylation status of *SERPINA1* in PBMCs is unknown. The aim of this study was to evaluate the methylation profile of the SERPINA1 promoter in PBMC. To this purpose PBMCs and serum were collected from healthy subjects (HS) (*n* = 75), including blood donors (BD) (*n* = 25), pregnant women at early pregnancy (EP) (*n* = 25), i.e., within the first trimester, and pregnant women at late pregnancy (LP) (*n* = 25), i.e., at the third trimester. DNA from PBMCs was treated with sodium bisulfite and PCR amplified for SERPINA1 gene promoter, followed by sequencing analyses. AAT serum levels were determined by ELISA test. *SERPINA1* was found hypermethylated in 58.7% of HS. The prevalence of *SERPINA1* hypermethylation was significantly higher in BD (68%) and EP (88%) than in LP (20%) (*p* < 0.01). The median serum AAT concentration was 1.07, 0.63, and 3.15 mg/ml in BD, EP, and LP, respectively (*p* < 0.05, BD and EP vs LP). This study indicates, for the first time, that SERPINA1 gene promoter is differentially methylated in PBMCs from HS. Likely, modulation of the methylation may be a novel epigenetic regulator mechanism of AAT expression in the PBMC of HS. Therefore, SERPINA1 gene promoter methylation may represent an epigenetic biomarker of PBMCs in healthy subjects.

## Introduction

Serine Protease Inhibitor-A1 (SERPINA1) gene encodes for Alpha1-AntiTrypsin (AAT), the second most abundant circulating serum protein (Ferrarotti et al., 2012). AAT is a protease inhibitor released from the hepatocytes constitutively (Miravitlles, 2012). It is primarily active in inflammation sites, where it plays a protective role for healthy cells adjacent to the inflamed tissue (Stockley, 2015). Indeed, AAT is delivered through the blood to the inflammation site, where it inhibits different proteases, in particular the elastase produced by neutrophils (Stockley, 2015). AAT activity is very high in the lower respiratory tract, where it provides more than 90% of the defenses against the elastolytic load of neutrophils (Yuan et al., 2018). AAT is also an acute-phase protein, and it is released in an inducible manner from hepatocytes and blood cells upon activation of *SERPINA1* inflammation-responsive promoter (Perlino et al., 1987). Levels of circulating serum AAT have been shown to be increased four-folds during infections, tissue injury and inflammation. Moreover, serum AAT levels increase physiologically four- to six-folds during the third trimester of pregnancy, and in elderly (Ferrarotti et al., 2012; Madar et al., 2013). How AAT could rise physiologically and maintain high levels for prolonged periods is not yet known. Regarding pregnancy, this is an important aspect given that lack of AAT increase exposes pregnant women and fetus to serious pathologies, such as pre-eclampsia and intrauterine fetal growth restriction (IUGR), which is the main cause of premature delivery and fetal mortality (Karowicz-Bilińska et al., 2007; Feng et al., 2016).

DNA methylation is one of the main epigenetic mechanisms for gene expression regulation (Attwood et al., 2002; Ohgane et al., 2008). The methylation of cytosine–guanine dinucleotides (CpGs) in the promoters of genes generally prevents the access of transcription factors (TFs), leading to transcription repression (Lea et al., 2018). Regions rich in CpGs, called CpG islands, are present in gene promoters and typically associated with gene transcription regulation through methylation (Lea et al., 2018). Differentially methylated regions (DMRs) are also involved in gene expression regulation, and are located upstream/downstream of transcription starting sites (Attwood et al., 2002; Ohgane et al., 2008). DMRs overlap with regions of variable CpG density and contain binding sites for different TFs, which can be differently methylated for controlling the gene expression in specific cells (Ohgane et al., 2008).

A recent family based epigenome wide association study (EWAS) in predominantly smoking adults, reported a positive correlation between hypomethylation at two CpGs in SERPINA1 gene promoter from peripheral blood mononuclear cells (PBMCs) and chronic obstructive pulmonary disease risk (Qiu et al., 2012). Counter wise, a more recent meta-analysis of tobacco-smoke exposed children and adults did not find correlations between SERPINA1 gene methylation from PBMCs and lung function (Beckmeyer-Borowko et al., 2018). These conflicting data raised the question whether methylation can or cannot affect SERPINA1 gene promoter in the PBMCs and prompted us to investigate in detail the methylation status of SERPINA1 gene promoter in PBMCs from healthy subjects (HS).

To this aim, the methylation profile of SERPINA1 gene promoter was investigated in PBMC from HS. The SERPINA1 promoter region located upstream of the transcription starting site containing eight CpGs and one binding site for TFs, was analyzed for the methylation. This promoter region includes the two CpGs found differentially methylated in the EWAS study (Qiu et al., 2012). PBMCs from three HS cohorts were collected: (i) blood donors (BD), (ii) pregnant women at early pregnancy (EP), and (iii) pregnant women at late pregnancy (LP). To assess the biological differences among HS cohorts, serum AAT level was evaluated in BD, EP, and LP.

## Materials and Methods

### Samples and DNA Isolation

Blood was from 75 healthy subjects (HS), including blood donors (BD) (*n* = 25), pregnant women at early pregnancy (EP), i.e., within the first trimester of gestation (*n* = 25), and pregnant women at late pregnancy (LP), i.e., at the third trimester of gestation (*n* = 25). Mean age ± standard deviation [SD] was 51.41 ± 11.07 years in BD, 32.62 ± 5.75 years in EP, and 34 ± 3.16 years in LP (BD vs EP, and LP, *p* < 0.0001; EP vs LP, *p* > 0.05). Blood samples were collected at the Clinical Analysis Laboratory, University Hospital of Ferrara, Ferrara, Italy. The hospital clinical records indicated that BD subjects and LP and EP women were in healthy conditions at the time of sample collection. Indeed, BD, EP, and LP samples belonged to healthy subjects, being blood analysis parameters in the normal index range. Blood samples from pregnant women were collected before surgical intervention from women underwent voluntary abortion (EP), and during the routinely controls of gestation (LP) (Tagliapietra et al., 2019, 2020). The County Ethical Committee of the University Hospital of Ferrara approved this study in accordance with the Declaration of Helsinki. Written informed consent was obtained from all subjects. PBMC and serum were separated by density gradient centrifugation (Tagliapietra et al., 2019). DNA was isolated from PBMCs using the QIAmp DNA Mini Extraction Kit (Qiagen, Milan, Italy) (Tagliapietra et al., 2019). After isolation, DNA concentration/suitability was assessed by spectrophotometric reading (NanoDrop 2000, Thermo Fisher, Monza, Italy), and by β-globin PCR-amplification (Tagliapietra et al., 2019).

### *SERPINA1* Methylation Analysis

SERPINA1 gene (GenBank: NG_008290.1) consists of three non-coding exons, Ia-bc, containing transcription starting sites for monocytes and macrophages (Ia and Ib) and hepatocytes (Ic) (Perlino et al., 1987). The 375 bp region upstream exon 1a (GenBank: NG_008290.1, HGNC:8941, position 4711–5085), containing eight CpGs, was investigated for methylation. Among the CpGs, CpG1, and CpG8 were found differently methylated in EWAS study (Qiu et al., 2012), and CpG6 overlapped with the CCGCCC-box consensus region (CG-box). DNA was treated with sodium bisulfite using the Epitect Bisulfite kit (Qiagen, Milan, Italy) (Rotondo et al., 2018). Samples were then purified using DNA purification columns (Epitect Bisulfite kit, Qiagen) and subsequently PCR amplified. SERPINA1 gene promoter was PCR-amplified using the F-5′-TTTTGGTTTAGTTTAGGATTTTGAGG-3′ and R-5′-ACCTACCAATTATTAATACCAAATCTATAC-3′ primers (Qiu et al., 2012). The PCR conditions were: 10 min of denaturation at 95°C, 40 cycles of 1 min at 95°C, 1 min at 65°C, and 2 min at 72°C, final extension for 5 min at 72°C. PCR products were directly sequenced using the automated ABI Prism 3730 × l-Genetic Analyzer (Applied Biosystems, Monza, Italy) (Rotondo et al., 2017). *SERPINA1* sequences showing more than or equal to 5 out of 8 methylated CpGs (≥50%) were considered hypermethylated (Rotondo et al., 2018).

### Serum AAT Levels Determination

The AAT levels in serum samples were determined using the Human alpha 1 Antitrypsin ELISA Kit (*SERPINA1*) (ab189579) (Abcam, Cambridge, United Kingdom), as reported (Hennawy et al., 2016). Diluted sera and antibody cocktails were incubated with AAT antibodies in pre-coated wells. After addition of 3,3′,5,5′-tetramethylbenzidine substrate, the colorimetric signal was read by optical density (OD) at a wavelength of 450 nm. The concentration of AAT was measured in duplicates, interpolated from the AAT standard curves (provided by the kit) and corrected for sample dilution (1:100,000), according to manufacturer’s instructions. The final AAT concentration in sera was reported as mg/mL.

### Statistical Analysis

The chi-square trend test with Yate’s correction was used to compare *SERPINA1* hypermethylation frequencies among BD, EP, and LP. Serum AAT values were analyzed with D’Agostino-Pearson test for normality, and values were compared using the non-parametric Mann–Whitney-*U*-test (Malagutti et al., 2020). Analyses were carried out using the GraphPad Prism 6.0 and the R package 3.5.0. *P* ≤ 0.05 was considered statistically significant.

## Results

### SERPINA1 Gene Promoter Methylation

**FIGURE 1:**
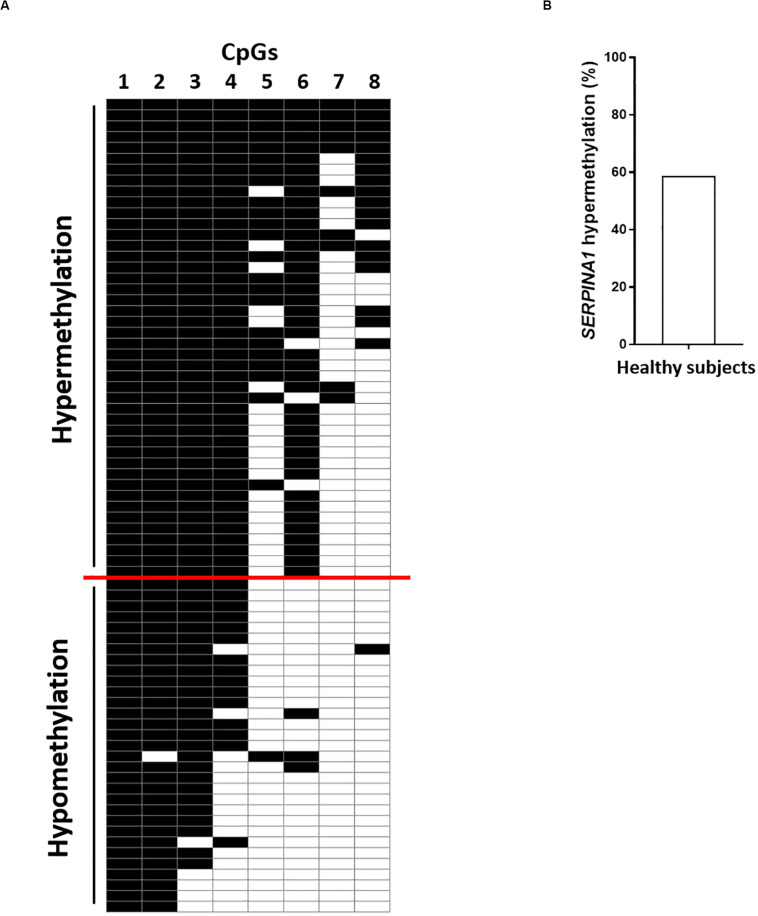
SERPINA1 gene promoter methylation analysis in healthy subjects (HS). **(A)** HS (*n* = 75) stratification according to the number of CpGs methylated (from top to bottom) in SERPINA1 gene promoter, i.e., hypermethylation and hypomethylation. Black boxes represent CpG methylation and white boxes CpG unmethylation. The CpGs within the SERPINA1 promoter are numbered across the top of the grid. Each row represents one sample analyzed by sequencing. Samples were considered hypermethylated when ≥5/8 CpGs (≥50%) were methylated. **(B)** Frequency of SERPINA1 gene promoter hypermethylation in HS (*N* = 75).

**FIGURE 2:**
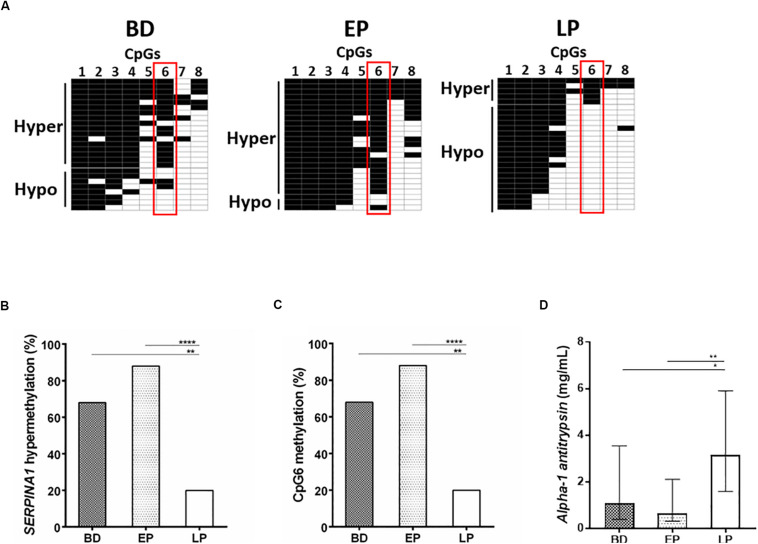
SERPINA1 gene promoter methylation analysis in healthy subjects (HS), including blood donors (BD) and pregnant women at early (EP), and late pregnancy (LP), and serum AAT levels determination in BD, EP, and LP. **(A)** BD (*N* = 25), EP (*N* = 25), and LP (*N* = 25) stratification according to the number of CpGs methylated (from top to bottom) in SERPINA1 gene promoter, i.e., hypermethylation and hypomethylation. Black boxes represent CpG methylation and white boxes CpG unmethylation. The CpGs within the SERPINA1 promoter are numbered across the top of the grid. Each row represents one sample analyzed by sequencing. Vertical red boxes highlight CpG6 across hypermethylated and hypomethylated samples. Samples were considered hypermethylated when ≥5/8 CpGs (≥50%) were methylated. **(B)** Frequency of SERPINA1 gene promoter hypermethylation in BD (*N* = 25), EP (*N* = 25), and LP (*N* = 25). **p < 0.01, ****p < 0.0001. **(C)** Frequency of CpG6 methylation BD (*n* = 25), EP (*n* = 25), and LP (*n* = 25). **p < 0.01, ****p < 0.0001. **(D)** Median (interquartile range [IQR]) AAT concentration in serum samples from BD (*n* = 25), EP (*n* = 25), and LP (*n* = 25). The AAT concentration in sera is reported as mg/mL. Concentrations were determined by ELISA test. *p < 0.05, **p < 0.01.

Using the bisulfite DNA treatment followed by PCR amplification and sequencing of the PCR products, the methylation profile of SERPINA1 gene promoter was assessed in PBMCs from HS (*n* = 75), including BD (*n* = 25), EP (*n* = 25), and LP (*n* = 25). PCR amplifications were efficiently obtained from all bisulfite-treated DNAs. The sequencing analyses showed that all cytosine residues adjacent to adenine, thymine, and cytosine in the original template were converted to thymine, indicating that the bisulfite conversion reaction was complete. HS samples stratification according to the number of CpGs methylated, from the highest to the lowest, showed *SERPINA1* methylation ranging between 5 and 8 CpGs methylated (hypermethylation) in 58.7% (44/75) PBMC samples and 1–4 CpGs methylated (hypomethylation) in 41.3% (31/75) (Figure 1, panels A,B). The CpG1 and CpG8 sites were found methylated in 100 and 25% HS, respectively. Samples stratification within the cohorts showed that the SERPINA1 gene promoter was hypermethylated in 68% (17/25) BD, 88% (22/25) EP, and 20% (5/25) LP (Figure 2, panels A,B). The difference in *SERPINA1* promoter hypermethylation prevalence between BD and EP vs LP was statistically significant (*p* < 0.01 and *p* < 0.0001, respectively, Figure 2, panel B). To assess if age may represent a possible cofounder factor between BD, EP, and LP (*p* < 0.0001) the methylation prevalence was tested in age-matched BD (*n* = 8, 37.13 ± 4.76 years; vs LP and EP *p* > 0.05) and no age-matched BD (*n* = 17, 58 ± 5.07 years; vs LP and EP *p* < 0.0001) (*p* < 0.0001). SERPINA1 promoter hypermethylation was detected in 62.5% (5/8) age-matched BD and in 70.6% (12/17) no age-matched BD (*p* > 0.05). These results indicate that age did not represent a confounder factor between BD, EP, and LP groups. The CpG6 site belonging to the CCGCCC-box consensus region showed methylation in 68% (17/25) BD, 88% (22/25) EP, and 20% (5/25) LP (BD vs LP *p* < 0.01; EP vs LP *p* < 0.0001, Figure 2, panels A,C).

### Serum AAT Levels

AAT levels were investigated by ELISA assay in serum samples from HS (*n* = 75), including BD (*n* = 25) and EP (*n* = 25) women, and LP (*n* = 25). Specifically, the median (interquartile range [IQR]) serum AAT protein concentration was 1.07 (0.39–3.54) mg/mL in BD, 0.63 (0.3–2.1) in EP and 3.15 (1.59–5.9) in LP (Figure 2, panel D). The difference in AAT concentration between BD vs LP, and EP vs LP was statistically significant (BD vs LP *p* < 0.05; EP vs LP *p* < 0.01, Figure 2, panel D). In age-matched BD and no age-matched BD the median serum AAT concentration resulted 1.11 (0.86–3.67) mg/mL and 0.59 mg/mL (0.37–3.02) (*p* > 0.05), indicating age to be not a confounder factor between BD, EP, and LP groups.

## Discussion

In the present study, the methylation profile of SERPINA1 gene promoter was investigated in PBMCs from three different HS cohorts, including BD, EP, and LP. Circulating AAT serum levels were investigated to assess biological differences among BD, EP, and LP groups. The LP group was particularly suitable to investigate impactful epigenetic changes in SERPINA1 gene, since systemic AAT increases physiologically four-six-fold during the third trimester of pregnancy.

SERPINA1 gene promoter was found hypermethylated in 58.7% (44/75) PBMCs from HS samples. To the best of our knowledge, this is the first study reporting hypermethylation of SERPINA1 gene promoter in PBMCs from HS. The frequency of SERPINA1 gene hypermethylation was significantly different among the three HS cohorts, showing higher prevalence in BD (68%) and EP (88%) compared to LP (20%). These results indicate that methylation (i) differentially occurs in SERPINA1 gene promoter, and (ii) may play a role in regulating the gene expression in PBMCs. In this context, promoter sequences interacting with the transcription factors, such as the regulatory consensus sequence CG-box, play a pivotal role in gene expression (Tu and Kiang, 1998). Selective methylation of a CpG within cis-acting regulatory elements in promoters at low CpG density can function as a mutation within the factor-binding site, preventing the binding to the TFs (Tu and Kiang, 1998). Our results indicate that the CpG6, belonging to the CCGCCC-box consensus region, was methylated in 68% SERPINA1 gene promoter from BD, 88% EP and 20% LP, suggesting that methylation at the CpG6 may be a transcription silencer in BD and EP. Interestingly, 100% of hypomethylated SERPINA1 samples from LP had the CpG6 unmethylated, further supporting methylation as a key player in differentially controlling SERPINA1 gene expression in PBMCs of HS. The two CpGs, CpG1, and CpG8, previously found to be hypomethylated in COPD smokers (Qiu et al., 2012), were found methylated in 100 and 25% of the SERPINA1 gene promoter from HS, respectively, supporting that changes in CpG methylation may occur in pathological conditions.

The circulating levels of AAT were investigated in serum samples from HS, including BD, EP, and LP. Immunological data showed that the median AAT concentration was 1.07, 0.63, and 3.15 mg/ml in BD, EP and LP, respectively (BD and EP vs LP, *p* < 0.05). These results are in agreement with previous data, reporting serum AAT concentration ranging 1.5–3.5 mg/mL in healthy individuals, including women at early pregnancy, and four/six-fold higher during the third trimester of pregnancy (Baron et al., 2012).

Taken together, our data show two different SERPINA1 gene methylation profiles in the PBMC of HS, one LP-associated hypomethylated, and one BD-EP-associated hypermethylated, suggesting methylation as a novel regulator of the AAT expression. Therefore, the modulation of methylation in the SERPINA1 gene promoter may allow AAT to be expressed in the PBMC of LP, and repressed in BD and EP. The reasons of this epigenetic regulation, and how it impacts in the LP women are currently unknown. However, DNA methylation results in long-term changes in gene expression (Hartley et al., 2013), which are crucial in controlling the longer term effects of stressors, such as oxidative stress and inflammation, typical conditions of the advanced pregnancy (Morris et al., 1998). In addition, several recent studies have highlighted immune regulatory roles of AAT, independent of protease inhibition, including induction of IL-10, suppression of plasma pro-inflammatory cytokines (Lewis et al., 2008; Persichini et al., 2016) and, interestingly, induction of immune tolerance in bone marrow transplants (Lewis et al., 2008). In this view, long-lasting profile of SERPINA1 gene hypomethylation may provide long-lasting increases in circulating AAT protein in LP women, allowing pregnancy stresses to be counteracted, and/or the maternal immune tolerance toward the fetus to be sustained for successfully pregnancy outcome. Indeed, lack of increase in ATT in the blood of pregnant women is associated with pregnancy complications, including the spontaneous abortion and premature rupture of membranes (Baron et al., 2012; Twina et al., 2012).

In conclusion, this study indicates that the SERPINA1 gene promoter is differentially methylated in PBMCs from HS, showing hypermethylation in BD and EP, and hypomethylation in LP. Likely, under specific physiological conditions, such as advanced pregnancy, modulation of the SERPINA1 gene promoter methylation may allow long-lasting expression of AAT. This epigenetic mechanism may allow *SERPINA1* to be expressed independently from inflammation/infection, enabling AAT to be risen physiologically and maintained at high levels for long time period during the late pregnancy. In this view, *SERPINA1* epigenetic regulation may guarantee AAT to be active not only as inflammatory/infection-induced protease inhibitor but also as immunomodulatory and tolerogenic protein, which could be the main AAT activities during the late pregnancy.

Therefore, methylation may be a novel epigenetic regulator mechanism of AAT expression in PBMC of HS. Further studies are needed to assess the direct link between AAT circulating levels and SERPINA1 promoter methylation in PBMCs.

## Data Availability Statement

The raw data supporting the conclusions of this article will be made available by the authors, without undue reservation.

## Ethics Statement

The studies involving human participants were reviewed and approved by the County Ethical Committee of the University Hospital of Ferrara in accordance with the Declaration of Helsinki. Written informed consent was obtained from all subjects.

## Author Contributions

FM, PR, and MT: study concept, design, and supervision. JR, LO-G, and RS: sample collection, analysis, interpretation of the data, and experiments execution. JR, RS, CL, CM, and GC: the data curation and interpretation, statistical analysis with software, and the data visualization. MT, MD, JR, and FM: writing of the manuscript and original draft preparation. MD, GC, PR, RP, FM, and MT: critical revision and discussion of the manuscript. MT and FM: funding acquisition and project administration. All authors contributed to the article and approved the submitted version.

## Conflict of Interest

The authors declare that the research was conducted in the absence of any commercial or financial relationships that could be construed as a potential conflict of interest.
